# Open-Label Placebo Treatment for Acute Postoperative Pain (OLP-POP Study): Study Protocol of a Randomized Controlled Trial

**DOI:** 10.3389/fmed.2021.687398

**Published:** 2021-11-05

**Authors:** Dilan Sezer, Matthijs de Leeuw, Cordula Netzer, Markus Dieterle, Andrea Meyer, Sarah Buergler, Cosima Locher, Wilhelm Ruppen, Jens Gaab, Tobias Schneider

**Affiliations:** ^1^Division of Clinical Psychology and Psychotherapy, Faculty of Psychology, University of Basel, Basel, Switzerland; ^2^Pain Unit, Department of Anesthesiology, University Hospital of Basel, Basel, Switzerland; ^3^Department of Spine Surgery, University Hospital of Basel, Basel, Switzerland; ^4^Division of Clinical Psychology and Epidemiology, Faculty of Psychology, University of Basel, Basel, Switzerland; ^5^Department of Consultation-Liaison Psychiatry and Psychosomatic Medicine, University Hospital Zurich, Zurich, Switzerland

**Keywords:** open-label placebo, acute postoperative pain, opioids, postoperative analgesia, placebo analgesia, lumbar interbody fusion

## Abstract

**Introduction:** Open-label placebos have been proposed as way of using long recognized analgesic placebo effects in an ethical manner. Recent evidence shows efficacy of open-label placebos for clinical conditions, but there is need for more research on open-label placebos in acute pain. In the treatment of acute postoperative pain, minimization of opioid related side effects remains one of the key challenges. Therefore, this study aims at investigating the potential of adding unconditioned open-label placebos to treatment as usual as a means of reducing opioid consumption and its related side effects in patients with acute postoperative pain.

**Methods and Analysis:** This is the protocol of an ongoing single site randomized controlled trial. The first patient was enrolled in May 2020. In total, 70 patients suffering from acute postoperative pain following dorsal lumbar interbody fusion are randomized to either a treatment as usual group or an experimental intervention group. The treatment as usual group consists of participants receiving a patient-controlled morphine pump. On day 1 and 2 post-surgery, patients in the intervention group receive, in addition to treatment as usual, two open-label placebo injections per day along with an evidence-based treatment rationale explaining the mechanisms of placebos. The primary outcome is measured by means of self-administered morphine during day 1 and 2 post-surgery. Several other outcome measures including pain intensity and adverse events as well as potential predictors of placebo response are assessed. Analysis of covariance will be used to answer the primary research question and additional statistical techniques such as generalized linear mixed models will be applied to model the temporal course of morphine consumption.

**Discussion:** This study will provide valuable insights into the efficacy of open-label placebos in acute pain and will potentially constitute an important step toward the implementation of open-label placebos in the clinical management of acute postoperative pain. In addition, it will shed light on a cost-efficient and patient-centered strategy to reduce opioid consumption and its related side effects, without any loss in pain management efficacy.

**Ethics and Dissemination:** The “Ethikkommission Nordwest- und Zentralschweiz” (BASEC2020-00099) approved the study protocol. Results of the analysis will be submitted for publication in a peer-reviewed journal.

**Clinical Trial Registration:** The study is registered at ClinicalTrials.gov (NCT04339023) and is listed in the Swiss national registry at kofam.ch (SNCTP000003720).

## Introduction

Placebo effects have been shown to have a clinically significant impact on subjective and objective health outcomes for a variety of somatic and mental disorders (1, 2). However, since the administration of deceptive placebo violates patients' right to autonomy [e.g., (3, 4)], alternative means of harnessing the placebo effect in an ethical manner—so-called Open-Label Placebos (OLP)—have been proposed and found to be effective in both healthy (5–10) as well as clinical populations [see (11, 12) for an overview].

Clinical investigation of OLP effects has mainly focused on chronic pain (13–17), allergic (18, 19), opioid use disorder (20), mental illness and psychosomatic symptoms (21–29). Evidence on OLP effects in acute pain on the other hand is limited, yet promising: Findings of two studies investigating the potential of Conditioned OLP (COLP) to reduce pain intensity and opioid dose in patients with spinal cord injury/polytrauma (30) and following spine surgery (31) suggest that COLP might also be effective in acute pain by showing reductions of opioid doses compared to Treatment As Usual (TAU). These results are supported by the findings of several experimental OLP analgesia studies in healthy populations (7, 8, 32–34). However, there is lack of investigations of unconditioned OLP in acute pain.

Patients undergoing dorsal Lumbar Interbody Fusion [LIF (35–37)] suffer from a great amount of acute postoperative pain (38) requiring intensive analgesia. Since Non-Steroid Anti-Inflammatory Drugs display a higher postoperative bleeding risk (39–42), opioids remain the primary systemic pharmacotherapy for intraoperative and postoperative analgesia. Therefore, minimization or prevention of opioid-related side effects is one of the key challenges of postoperative analgesia in dorsal LIF patients.

In the light of these current challenges in postoperative pain management in dorsal LIF patients and the promising results of above mentioned OLP studies, adding OLPs to an opioid-based TAU could provide a means of harnessing analgesic placebo effects (43–45) in acute postoperative pain. This approach could lead to a reduction in postoperative opioid consumption and less opioid-related side effects, without any loss in pain management efficacy.

In this randomized controlled trial, TAU mainly consists of a patient controlled, morphine-based analgesia. Patients in the OLP group will receive additionally two saline injections a day, which will be disclosed openly to the patients as placebo injections. By choosing injections instead of pills, we hope to maximize the OLP response, as it has been shown that placebo effects are bigger the more invasive a treatment is (46, 47). In addition, the setting of this study is suitable to test for the first time OLP injections as venous access is already established due to the postoperative setting.

By adding OLP injections to the TAU this study is the first to investigate OLPs potential to reduce morphine consumption in acute pain without conditioning and thus by solely relying on expectancies induced by verbal suggestions and previous experiences (48–51). We hypothesize that patients receiving the OLP injections in addition to TAU will administer themselves less morphine. Furthermore, the study design also allows to assess the effect of OLP injections on morphine desire (i.e., clicks on the patient-controlled analgesia pump exceeding the maximum of allowed morphine consumption), self-reported pain intensity, interference of pain with different areas of functioning, amount of requested rescue analgesics, number of reported side effects, and length of hospitalization. Finally, this study provides the opportunity to investigate the influence of several psychological factors associated with the OLP response.

## Methods and Analysis

### Study Design

**Figure 1:**
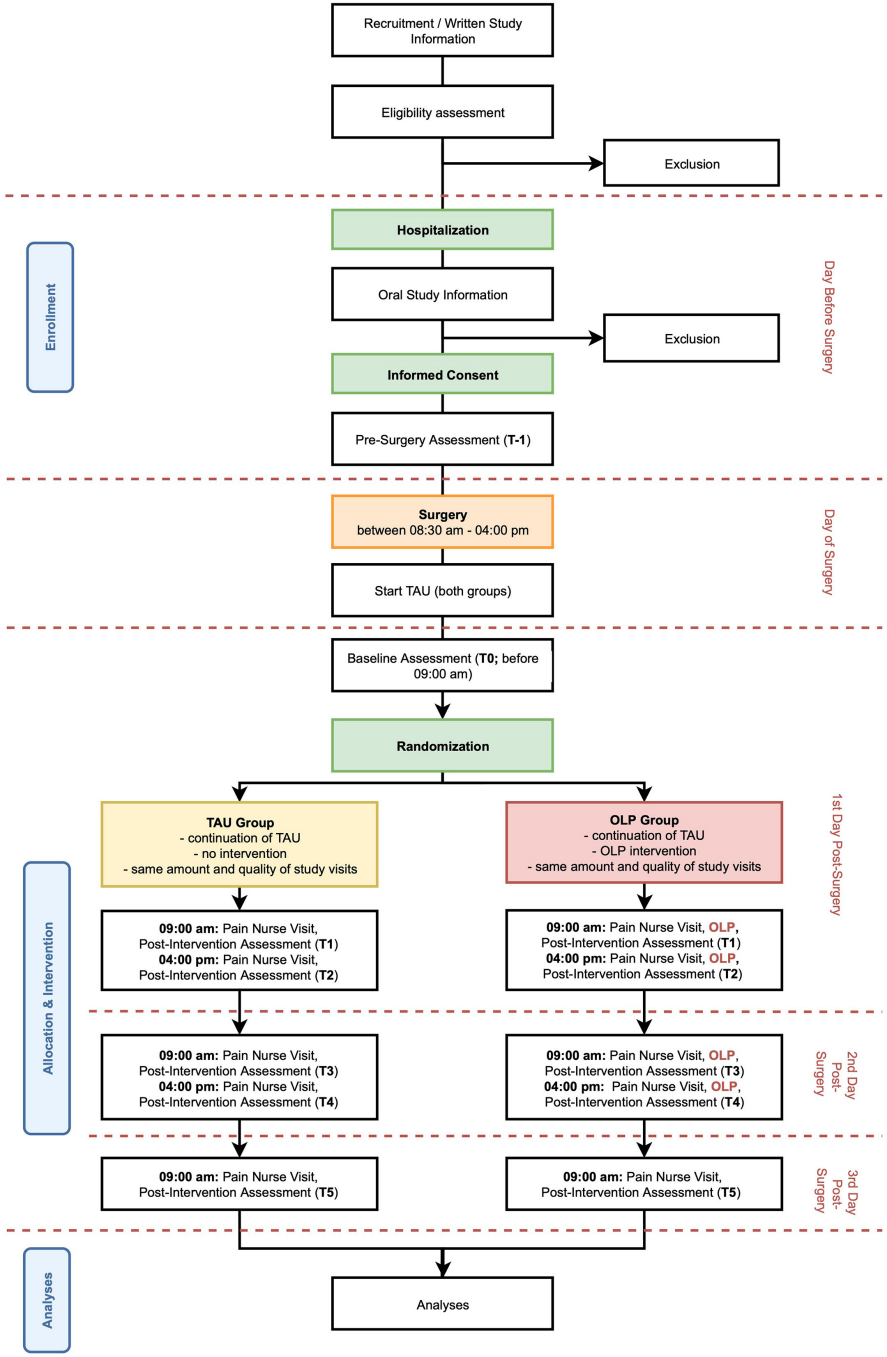
Study design and flow. TAU, Treatment as usual; OLP, Open-label placebo.

This ongoing assessor blinded study is designed as a single center, randomized controlled trial with a parallel group design using block randomization with a 1:1 allocation, comparing an OLP intervention group and a TAU control group (see Figure 1). The first participant was enrolled and randomized in May 2020, and the study is expected to be concluded by December 2023 with the planned inclusion of 70 study participants, this corresponds to a recruitment rate of two patients per month. The study is being conducted at the University Hospital of Basel by the Pain Unit and the Faculty of Psychology at the University of Basel in collaboration with the Department of Spinal Surgery of the University Hospital of Basel.

### Study Population

The study population consists of patients receiving elective dorsal LIF surgery at the University Hospital of Basel.

#### Inclusion Criteria

All patients scheduled for elective surgery with decompression and posterior fusion of the lumbar spine are potential study candidates. To facilitate an acceptable comparability between the study patients regarding wound surface and surgical trauma, operation procedures acceptable for inclusion are closer specified. Patients can be included if:

- The primary operation includes only the segments of the lumbar spine (L1-L5), plus the first sacral segment (S1).
- In this defined area, fusions of up to two levels (for example: L1-L3) are allowed.
- Additional decompressions are allowed, if performed at the segments of the stabilization or the direct proximate segments above or below, if the procedure does not exceed the segments L1-S1.

In addition, participants also have to fulfill all of the following inclusion criteria for study eligibility:

- 18 years or older.
- German speaking.
- Able to understand the study and its outcome measures.
- Able to provide Informed Consent (IC).

#### Exclusion Criteria

The presence of any one of the following exclusion criteria leads to exclusion of the participant:

- Known chronic pain, which is unrelated to the problem targeted by the surgery.
- Known neuromuscular disease.
- Known mental disorders.
- Known drug or massive alcohol intake or intake of other psychoactive substances.
- Known kidney or liver disease (glomerular filtration rate <30).
- Contraindications to the class of drugs under investigation, e.g., known hypersensitivity or allergy to the investigational product.
- Parallel participation in another study with investigational drugs.
- More than 30 mg/day (equivalent dose of oral morphine) preoperative opioid consumption.

Participants may continue to use their regular medication (e.g., hypertension, diabetes, etc.; cf. Concomitant Treatments). However, participants should not change the routine or dosage during the trial, if possible. Any medication intake and changes are assessed thoroughly.

### Recruitment and Screening

Inclusion and exclusion criteria of patients scheduled to receive dorsal LIF are being verified by using the electronic hospital records and double-checked by physicians. If patients are eligible for participation, they receive written and oral information about the study provided by study team members. After hospital admission on the day before surgery, eligibility is assessed again, open questions are answered, and IC is obtained. After IC patients fill in several questionnaires (see Table 1 for an overview of study assessments).

**Table 1:** Assessment timeline.

		**Screening**	**Pre-Surgery**	**Baseline**	**Intervention**					**Completion of Each Participants Data**
		**Screening**	**T-1**	**T0**	**T1**	**T2**	**T3**	**T4**	**T5**	
**Activity/Variable**	**Duration in minutes**			**before 09:00a.m**.	**09:00a.m**.	**04:00p.m**.	**09:00a.m**.	**04:00p.m**.	**09:00a.m**.	
Patient information and informed consent	15	x								
In-/Exclusion criteria	1	x								
Socio demographics	1		x							
Medical History (i.e., Medication at hospital admission analgesic consumption)	0		x							
Preoperative anxiety	2		x							
Pain catastrophizing	3		x							
Depression	2		x							
Placebo beliefs and understanding	1		x							
Opioid beliefs	2		x							
Comprehensive pain assessment	5		x	x			x		x	
Back and leg pain intensity at rest	1		x	x	<———————————————————————–>^*^					
Back and leg pain intensity while walking	1		x	x		x		x		
Expectancy of pain relief	2		x	x	x	x	x	x	x	
Randomization	0				x					
Check and if needed adjust PCA	2				x	x	x	x	x	
Morphine consumption and desire	0				x	x	x	x	x	
Intervention (OLP group only)	2				x	x	x	x		
Intervention credibility	3/1				x				x	
Disappointment (TAU group Only)	1								x	
Open qualitative questions	3								x	
Side-effects-related medication request	0									x
Concomitant medication/interventions	0									x
Length of hospitalization and details on the surgery	0									x
Rescue medication request	0									x
estimated duration for patients (Min)		16	20	9	10	8	12	8	15	0

*PCA, Patient controlled analgesia; TAU, Treatment as usual; OLP, Open-label placebo*;

* *continuous assessment every 2 h*.

### Randomization and Treatment Allocation

A random treatment allocation was generated by an independent investigator. Treatment assignments were drawn from a computer-generated random number sequence. Sequentially numbered opaque envelopes containing treatment allocation are used to assign participants to either the OLP group or the TAU group. In order to guarantee equal distribution of conditions, randomization was performed in blocks of ten, leading to five TAU and five OLP participants for each block of 10 participants.

Treatment allocation occurs prior to the first study visit on day 1 post-surgery (i.e., T1). The pain nurse performing the subsequent study visit opens the corresponding envelope and reveals the treatment assignment by letting the patient know the group to which they have been assigned to.

### Blinding Procedures and Other Methods of Minimizing Bias

Blinding participants is not possible, as it is an open-label trial. However, the primary outcome of interest (morphine dose) is never explicitly mentioned to the participants of both groups. They are only informed about the non-specific therapeutic benefits that are associated with placebo analgesia.

As treatment allocation occurs after baseline assessments are completed, study pain nurses are blinded up to day 1 post-surgery (T1). Team members responsible for assessments of outcomes subsequent to the pain nurse visits are blind to the group assignment during the whole study. Furthermore, hospital staff not involved in the study (e.g., ward nurses and doctors, who assess some side effects) is not aware of the group allocation and thus blinded.

As disappointment may result from allocation to the control group (52) which can lead to nocebo effects (53, 54), participants of the control group are reminded of the importance of the control group after randomization (cf. Supplementary Material for exact wording). Disappointment in the control group is also assessed at the end of the trial (T5).

Moreover, manualized instructions are used during all study specific contacts and study team members are instructed to treat participants in both groups equally supportive with empathy and warmth. In addition, mostly validated questionnaires are used in this study. If no validated German version of a particular questionnaire was available, we translated the questionnaire using back translation following the procedure proposed by Beaton, Bombardier et al. (55).

### Study Visits and Study Procedures

After inclusion of patients into the study, there are several study visits (cf. Figure 1 for an overview of study timeline). Procedures and timeline of visits are described in the following. An overview of all assessments made at each visit can be found in Table 1.

- **T-1:** After the IC is signed (cf. 3.3 Recruitment and Screening) patients answer a series of questionnaires including patient's preoperative anxiety, depression, beliefs regarding placebos and opioids, pain and postoperative pain expectancies.
- **T0:** Before 09:00 a.m. on day 1 post-surgery, a study team member visits the patient. Baseline assessments of current pain are made.
- **T1:** At circa 09:00 a.m. on day 1 post-surgery, a specialized pain nurse checks and if needed adjusts the PCA pump and assesses morphine consumption since installation of the pump on the day of surgery. The pain nurse reveals the treatment allocation to the patient. In addition, if the patient is in the OLP group, the experimental intervention is performed (cf. Intervention for more information on the intervention). After the pain nurse visit, patients of both groups answer again questions regarding treatment expectancy under supervision of a study team member.
- **T2:** At circa 04:00 p.m. on day 1 post-surgery, the pain nurse checks and if needed adjusts the PCA pump again. Morphine consumption since the last study visit is assessed. In addition, if the patient is in the OLP group, the experimental intervention is performed again. After the pain nurse visit, the patient answers again several questions regarding pain and pain expectancy under supervision of a study team member.
- **T3:** Same procedure as T2 at circa 09:00 a.m. on day 2 post-surgery.
- **T4:** Same procedure as T2 at circa 04:00 p.m. on day 2 post-surgery.
- **T5:** Same procedure as T2 but without intervention in the OLP group, at circa 09:00 a.m. on day 3 post-surgery.

### Intervention

#### Control Intervention (Treatment as Usual)

In this study, the TAU group serves as a control group. After randomization, all participants in this group continue TAU and concomitant medication and have the same amount and quality of contacts with the study team. However, participants of this group do not receive any intervention.

TAU consists in both groups of:

- **Basic analgesia:** 3 grams of Paracetamol per os a day
- **Patient controlled analgesia (PCA):** Patient-controlled morphine pump configured to release a maximum of 2 mg of morphine every 12 min; dosage can be adjusted in the course of treatment if rescue medication is not effective enough or if side effects occur
- **Rescue medication:** 1,000 mg of Metamizol, maximum every 6 h or in case of allergy to Metamizol 400 mg of Ibuprofen, maximum every 6 h.

#### Experimental Intervention

All participants in the OLP group also receive TAU as described above. In addition, they receive an experimental OLP intervention. This intervention consists of two components: An evidence-based treatment rationale and OLP injections.

##### Treatment Rationale

The idea of delivering an evidence-based treatment rationale alongside with the OLP injections has been driven by the known underlying mechanisms of deceptive placebo analgesia (e.g., treatment expectation, classical conditioning). Thus, eliciting a positive treatment expectation (10) by informing the patient about the evidence supporting OLPs as well as assumed mechanisms of action (e.g., classical conditioning) has been thought to be an incremental component of OLP interventions (56, 57). However, evidence on the necessity to deliver an evidence-based treatment rationale alongside the OLP intervention as introduced by Kaptchuk, Friedlander et al. (22) is mixed: On the one hand, findings of different OLP studies including our own study in experimental pain (7) suggest that an evidence-based rationale is indispensable in OLP efficacy (10). On the other hand, there have also been investigations showing no additional improvement when a treatment rationale was delivered alongside placebo administration: For example, allergic symptoms were similarly reduced even when pills were given without further explanation (19). In line, our recent study on OLP analgesia in healthy male adults (32) showed a comparable effect on pain reduction in both a short education group as well as in a detailed education group. This result is of great importance, because the possibility of providing OLPs with a short education makes them feasible in clinical practice.

Despite the conflicting evidence base, patients in this study receive an evidence-based treatment rationale (cf. Supplementary Material) prior to the administration of the first OLP injection (T1). This treatment rationale is thought to increase patients' perceptions of practitioner competence and empathy (58, 59) as well as the plausibility of the placebo intervention treatment (60), which in turn may enhance placebo effects. Thus, the treatment rationale is perceived as an incremental component of the OLP intervention and is therefore not given to the control group.

The rationale states clearly the fact that the placebo injections are inactive (inert) and contain only saline (i.e., salt and water). Further, based on previous OLP studies (22), it contains the following discussion points, which have been adapted to refer to the specific placebo analgesia and study context (i.e., adding treatment expectation, a second placebo analgesia mechanism and dismissing the “original” discussion point on the importance of adherence):

- Placebo effects of OLP can be **powerful** in some patients, especially in analgesia.
- **Treatment expectations** are found to be an important mechanism in placebo analgesia.
- In response to placebos the body can **automatically** release **endogenous opioids** which are targeting the pain, experienced due to the surgery.
- A **positive attitude** is helpful but is not absolutely necessary.

At every subsequent placebo application (T2, T3, T4), the patient is reminded of the inertness of the injection and that OLPs might help with regulating pain (cf. Supplementary Material).

##### OLP Injections

Five milliliter syringes containing 5 ml of saline 0.9% are used as placebo. The syringes are labeled with a blue “Placebo” sticker which is visible to the patients. These placebo injections are given twice a day (at 09:00 and at 04:00 p.m.) on day 1 and 2 post-surgery (i.e., patients receive a total of four placebo injections). The injections are administered intravenously; the access is the same as for TAU. It is ensured that patients watch the injection. Since the intervention is delivered by pain nurses, treatment adherence is warranted.

It is important to note that saline and its effects are not the product under investigation, but the presumed psychological mechanisms of the therapeutic procedure—the act of receiving a treatment and a plausible explanation alongside—is expected to have the most important impact on pain perception of patients. Therefore, saline could be replaced by any other carrier solution without analgesic properties (e.g., Ringer lactate).

#### Dose Modifications

TAU can be modified, if necessary, according to this scheme:

- **Analgesia, including rescue medication, is not sufficient and opioid-related side effects are tolerable:** The PCA pump can be adjusted, so that 2 mg of morphine can be administered every 8 min. A limit of 14 mg morphine per hour is set.
- **Opioid-related side effects are not tolerable, and analgesia is sufficient:** The PCA pump can be adjusted, so only 1 mg of morphine can be administered every 12 min. Increasing the lock-out time to 15 min, with or without a bolus reduction, is also possible.
- Treatment of opioid related side effects according to in house standards (i.e., antiemetic's, laxatives) is possible at any time.

Dose modifications beyond these defined adjustments lead to study discontinuation. Discontinuation or modification of the experimental intervention (cf. Experimental Intervention) and its dose is not intended. Premature ending of the intervention is being encouraged if a given participant reports serious deterioration, which is not to be expected.

#### Concomitant Treatments

There are no restrictions regarding concomitant interventions or treatments (e.g., opioid-related side effects medication, or physiotherapy), except for simultaneous participation in other studies with investigational drugs. Concomitant interventions or treatments are regularly documented within hospital standard documentation routines. After study completion of each patient, data is extracted from the electronic patient record of the patient and entered into the electronic case report form (eCRF; cf. Data Collection, Management and Storage).

### Outcome Measures

A detailed timeline of all outcome assessments is provided in Table 1.

Due to the characteristics of the study population (i.e., not being digital natives), self-reported bi-hourly assessments of pain intensity is being delivered in paper-pencil format. All other assessments are administered digitally on a tablet-PC and are supervised by a study team member. Thus, adherence to all assessments with exception of the bi-hourly assessments of pain intensity is warranted.

Primary and secondary outcome measures will be presented as means with SD if appropriate.

#### Primary Outcome

Primary study outcomes are assessed by means of the cumulative dose (i.e., total amount) of self-administered morphine within 48 h starting on day 1 post-surgery and ending on day 3 post-surgery.

#### Secondary Outcomes

Secondary outcomes comprise the following:

##### Morphine Desire Rates

Morphine demand behavior is measured by the total number of unsuccessful clicks on the PCA pump, allowing to quantify participants desire of morphine, exceeding the maximum amount they can administer themselves.

##### Pain Intensity at Rest and While Walking

Following the recommendations made by the Initiative on Methods, Measurement, and Pain Assessment in Clinical Trials [IMMPACT (61)] back and leg pain intensity at rest and while walking are measured separately several times a day (cf. Table 1) by four eleven-point Numeric Rating Scales (62, 63).

##### Comprehensive Pain Assessment and Patients' Perception of Postoperative Pain Management

Comprehensive pain intensity, frequency, duration, and interference as well as side effects of pain medication are assessed by the German version of the International Pain Outcomes Questionnaire (64). To be able to administer the questionnaire several times, the time period statements (i.e., “since your surgery”) was changed to “the last 24 h” (cf. Supplementary Material for information on additional minor adaptions).

##### Requested Rescue Analgesics

Amount of administration, dosage and time of administration are assessed for the time period of T0–T1 (i.e., baseline consumption of rescue analgesics) and T1–T5 (i.e., post-intervention consumption of rescue analgesics).

##### Opioid-Related Side Effects

Nausea, vomiting and constipation (i.e., stool frequency, vomiting and amount of delivered laxatives and antiemetics) as well as serious adverse events (e.g., oxygen desaturation) are assessed within the routine hospital documentation for the time period of T0–T1 (i.e., baseline rate of opioid-related side effects) and T1–T5 (i.e., post-intervention amount of opioid-related side effects). Other opioid-related side effects (e.g., nausea, drowsiness, itching, and dizziness) are assessed within the International Pain Outcomes Questionnaire (i.e., each morning post-surgery).

##### Length of Post-surgery Hospitalization

Data is collected upon participants trial completion.

#### Other Variables of Interest

Other variables of interest are:

##### Expectancy of Pain Relief

Expectancy of pain relief are assessed separately for leg and back pain with an eleven-point Likert-scale each (cf. Supplementary Material). Thereby, the influence of the OLP-intervention on patient's expectancy of pain relief as well as its effects on morphine consumption are investigated.

##### Intervention Credibility

After the first OLP injection and at the end of the trial the OLP group is asked about the credibility of the OLP intervention (for details cf. Supplementary Material).

##### Depression

In order to assess the influence of depressive mood on the placebo response (65, 66), the depression scale of the German version of the Patient Health Questionnaire (67) is administered on the day before surgery (i.e., T-1). This questionnaire assesses depressive symptoms according to the criteria of the fourth edition of the Diagnostic and Statistical Manual of Mental Disorders (68).

##### Pain Catastrophizing

High levels of pain catastrophizing are associated with a heightened pain experience and appear to contribute to the development of chronic pain in patients suffering from postoperative pain (69). To assess the influence of pain catastrophizing on the outcomes of this study, the German version of the Pain Catastrophizing Scale (70) is administered prior to study start (i.e., T-1).

##### Preoperative Anxiety

Preoperative anxiety is known to influence postoperative pain levels (71) and is thus assessed, using the German version of the Amsterdam Preoperative Anxiety and Information Scale.

##### Placebo Beliefs and Understanding of Patients

Placebo beliefs and understanding of patients are assessed by two different means:

- In order to assess the influence of patients pre-existing placebo beliefs on the OLP effect, a translation of a four-item questionnaire introduced by Leibowitz, Hardebeck et al. (6) is administered.
- Placebo understanding is assessed by the first two items of a questionnaire introduced by Fassler, Gnadinger et al. (72) which assesses responders' attitudes regarding non-specific therapies. The first two items specifically assess the placebo understanding of responders.

##### Opioid Beliefs of Patients

In order to assess the influence of patients pre-existing opioid beliefs on the amount of morphine consumption (73), we apply a translation of a ten-item questionnaire assessing beliefs regarding opioids which has been introduced by Lai, Dalton et al. (74).

#### Safety Outcomes

No specific adverse events, serious adverse events or side effects due to the placebo intervention are expected. Moreover, if the OLP intervention provided in this study can reduce morphine intake in some patients, side effects due to TAU can potentially be decreased. Thus, patients in the intervention group might even be exposed to less harm, than patients in the TAU group. Side effects due to TAU are therefore assessed as a secondary outcome. Furthermore, only serious adverse events are assessed.

### Data Collection, Management, and Storage

For data collection and management of participants responses at study visits, the secure web application REDCap (75) is used as eCRF. The system is hosted by the Center for Scientific Computing of the University Basel (sciCore). Due to password protection, only authorized personal is able to enter the system and to view and edit data. Entries and actions within the application are marked with a date and time stamp and the name of the respective study team member who locked into the system. Double data entry is performed in REDCap to digitalize all source documents. In addition, data preparation of PCA protocols is done by two independent study team members. All data entries in REDCap are deidentified. Regular back-ups of study data take place and back-ups are stored on secure webservers of the University Hospital of Basel.

### Sample Size

Reported effects sizes for OLP effects in clinical and sub-clinical trials are generally medium to large (11, 12) with overall Standardized Mean Differences (SMD) of 0.72–0.88. The confidence intervals reported in the larger meta-analysis by von Wernsdorff, Loef et al. (12) suggests that there is substantial variability in the observed effect sizes among the different studies, ranging from SMD 0.39 to 1.05. Therefore, using the statistical software G^*^Power we conducted a conservative power calculation on the basis of an F-test for an ANCOVA for two groups. This analysis showed that we would need a sample size of *n* = 84 for a power of 0.8 to detect a medium effect size of *d* = 0.55 with a one-sided alpha-level of 0.05 when disregarding any covariates. We then estimated by which factor the residual variance in our primary outcome variable would decline as a consequence of the additional explained variance by our two covariates (see description below). We thereby assumed that they would explain an additional 25 percent of the variance of the outcome as an upper limit (*r*^2^ = 0.25). This assumption is based on a suggested correlation of *r* = 0.5 between the baseline morphine consumption and the post-randomization consumption. In terms of the reduced residual variance this would lead to a decline by a factor of 1– *r*^2^ = 0.75 and as a consequence to an increase of the effect size *d* by 1/sqrt(0.75) = 1.15 yielding an expected effect size of *d* = 0.635. This in turn would reduce the sample size necessary as computed above to *n* = 64.

Based on these calculations and considerations, we decided to enroll a total sample size of 70 (i.e., 35 per group) which takes a drop-out rate of c.10% into account. This sample size is comparable with previous two-armed clinical OLP studies which have found medium to large effect sizes [e.g., (13): *n* = 83, *d* = 0.76, (22): *n* = 80, *d* = 0.79].

### Statistical Analysis

The primary research question of this study is whether there is a difference in the total amount of morphine consumed over the course of the intervention period (i.e., across 48 h) between the two groups. In order to answer this question, we will compare the total amount of consumed morphine across the two groups using a one-way ANCOVA. Baseline morphine consumption (i.e., consumption prior to randomization) and patients' history of morphine consumption [calculated as morphine equivalent dose by the in-house opioid calculator (76)] prior to study start are the two covariates, and treatment group the between subject factor. We expect that the OLP intervention group will show significantly lower morphine consumption over the course of 48 h (T1–T5) in comparison to the TAU control group.

Regarding our primary outcome, we are in addition interested in answering the following questions:

1. **Do the temporal fluctuations of morphine consumption differ over the course of 48 h between the two groups?** To answer this question, we will calculate the amount of morphine consumption for intervals of 12 min (corresponds to the lock-out period of the PCA) starting at the time of the first study visit (i.e., start of the intervention period) for a total of 48 h. This yields a total of 240 intervals each indicating if morphine was consumed within this time period or not. We will then calculate the Root Mean Square of Statistical Differences (RMSSD) indices for each patient as a measure of variability over time and compare them between the two groups using an ANCOVA with baseline RMSSD of morphine consumption as covariate.
2. **How does the course of consumption of the two groups evolve over time?** This question will be answered by using again the data with the 12 min intervals and by performing Generalized Linear Mixed Model (GLMM) analyses with morphine consumption (yes/no) as dichotomous dependent variable, group as between subjects factor, time as within subjects predictor, including the interaction time x group. The predictor time may be included as a linear term or, depending on the observed temporal course, as a curve-linear term. In case of a more complex temporal pattern, the use of Generalized Additive Mixed Models (GAMMS) might be useful as these models allow for a smoothing function to more flexibly model the temporal course.

Analyses of secondary outcomes (e.g., morphine desire rates, pain intensity, etc.; cf. Secondary Outcomes) will also focus on group differences, whereby covariates (such as baseline variables) will be included in the statistical model if they are known to be predictive of the respective outcome. In case no covariates are included, we will use a *t*-tests instead of an ANCOVA to analyze group differences. Furthermore, explorative regression analysis of potential predictors (e.g., preoperative anxiety, placebo beliefs, etc.) of morphine consumption will be performed.

In case of missing data, multiple imputation will be adopted prior to the analysis. All analysis will be performed using RStudio for Mac. Any deviation from the here reported statistical plan will be described and justified in the final report, as appropriate.

### Monitoring

The study is monitored for quality and regulatory adherence by an independent monitor of the University Hospital of Basel. The monitor verifies the qualification of the investigators and study team members and monitors sound and appropriate documentation. In addition, monitoring visits serve to approve that:

- The study is conducted according to the study protocol and within the specified time frame.
- Data is collected accurately and completely documented in REDCap and the source documents.
- The intervention medication (placebo injections) is correctly prepared, dispensed and accounted for.
- Side effects are correctly defined, assessed and documented.

## Discussion

Despite intense research during the last 50 years, adequate pain management—especially in the postoperative phase—is still a challenge. The available selection of pharmacologic agents is limited, and their clinical use is often restricted by their (dose dependent) side effects. Even more, high dosages of analgesics can harm the patient. Respiratory failure, due to opioid overdose, or gastric toxicity of non-steroidal anti- inflammatory drugs (40), especially in the most vulnerable (old and multi-morbid patients) are only two examples. Furthermore, since the opioid crisis in the US (77) and raising opioid prescriptions even in Switzerland and worldwide (78, 79), there is a great interest in developing new medications and treatment strategies to reduce acute pain, analgesic demands and thereby improve patient's safety.

OLPs hold the potential of using placebo effects in an ethical manner. This is of special interest in the area of pain where placebo effects and placebo responses have been long recognized and are well investigated. OLPs have been shown to be effective in some clinical populations [see (11, 12) for an overview], e.g., in chronic low back pain (13, 15–17). In addition, there are promising results by several experimental OLP analgesia studies (7, 8, 32–34). However, concerning OLP effects in acute pain, there is only limited evidence. So far, only two Randomized Controlled Trials (RCTs) have investigated OLP in acute pain (30, 31). Both of these studies have used an OLP conditioning paradigm in order to reduce opioid doses compared to TAU. To our knowledge, the present study is the first RCT investigating an OLP intervention without conditioning in the clinical management of acute postoperative pain. Results of this trial will thus inform about the efficacy of adding OLP to TAU as a potential means to reduce opioid doses, but also about the feasibility to integrate OLPs in the management of acute postoperative pain.

A main strength of our study design is the use of the PCA pumps. The pump enables the patient to self-administer 2 mg of morphine every 12 min which allows us to measure the exact consumption of morphine. During the 12 min lock out time it is not possible to administer a second bolus, but each click on the PCA will be saved by the pump. Therefore, we are also able to assess the amount of morphine desire (i.e., number of clicks on the pump without bolus application). Thus, using the PCA as primary outcome measure allows to measure continuously and indirectly patient's pain perception in addition to the self-reported pain intensity ratings. This indirect measure addresses one of the primary shortcomings of previous OLP studies which mostly rely on subjective self-reported questionnaires. In addition, measuring morphine consumption as indirect indicator of participant pain can minimize reporting bias (e.g., wishing to please the experimenter), when comparing to subjective pain ratings. Furthermore, the continuous nature of our primary outcome can provide information on how long OLP effects can last. Beyond that, finding adequate control groups has been identified as an issue in previous OLP trials (11). We address this problem by offering the same amount and quality of contacts with the study team in the control as well as the intervention group. In addition, the TAU group allows to control for the natural course of postoperative pain, regression to the mean, and other biases inherent to clinical trials (80). Finally, the assessment of many different questionnaires including attitudes and experiences of participation enables us to investigate underlying mechanisms and factors influencing the placebo response.

The chosen design and setting of this study entail some limitations. Firstly, due to the specific study population of dorsal LIF patients the results might not be generalizable to all patients suffering from acute postoperative pain. Secondly, although we tried to implement blinding procedures as much as possible, due to the nature of OLPs, reporting bias cannot be ruled out. Thirdly, we are aware that giving a rationale only to the intervention group differs from procedures of prior studies investigating OLPs (19, 21–23), which raises questions regarding balanced patient-provider interaction time across conditions. However, TAU in the postoperative pain care normally does not include an OLP rationale. In addition, providing the rationale might lead to disappointment in the control group and even increase the possible difference between OLP and TAU. Finally, as the sample size is relatively small and the intervention phase is short, more investigation will be needed to allow clinical recommendations.

To sum up, this study strives to contribute to the young research field of OLP, which aims at elaborating ways of harnessing placebo effects ethically in clinical practice and thereby enabling a new cost-efficient way of evidence-based patient-centered medicine. It is the first study to investigate OLP effects without conditioning in a clinical sample suffering of acute pain and might therefore be of great importance in answering the question whether the knowledge we have about deceptive placebos in acute pain can also be applied to OLP. Furthermore, due to its interdisciplinary set up at the University Hospital of Basel, this study contributes to the process of raising awareness about placebos in the clinical day to day live and contributes to answering questions about the real-life applicability of placebo treatments in clinical practice.

## Ethics and Dissemination

This study is carried out in accordance with the protocol and principles enunciated in the current version of the Declaration of Helsinki (81), the guidelines of Good Clinical Practice (GCP) issued by the International Council for Harmonization of Technical Requirements for Pharmaceutics for Human Use [ICH (82)], the ISO norm 14155 [International Organization of Standardization (83)], the ISO norm 14971 (84), and the Swiss law and Swiss regulatory authority's requirements. In compliance with our in-house ethical guidelines, patients received no compensation for taking part in the study.

### Confidentiality

Data will be handled confidentially, be protected and encoded. Participants' confidentiality will be maintained at all times. Direct access to source documents will be permitted for purposes of monitoring, audits, and inspections, however while respecting medical secrecy and refraining from divulging participants' identity. Co-investigators and study team members (i.e., pain nurses and master students) will have access to the protocol, datasets, and statistical codes during and after study conduct.

### Access to Data

Only investigators and study team members will have access to relevant data on the computer system of the University Hospital of Basel.

### Dissemination Policy

The results of the planned analyses will be published in a peer-reviewed journal. Talks at conferences and other occasions (e.g., teaching) are also planned.

## Ethics Statement

The study protocol for this study was approved by “Ethikkomission Nordwest- und Zentralschweiz” (BASEC2020-00099) on April 4, 2020. Trial and protocol design were developed according to the Standard Protocol Items: Recommendations for Interventional Trials (SPIRIT) guidelines (85). Substantial protocol amendments are only implemented after approval of the competent ethics committee. Patients interested in study participation are provided with sufficient oral and written information for an informed decision concerning participation. IC is only obtained if participants meet the inclusion criteria and thus are over the age of 18, can understand the study and are able to provide IC. Withdrawal from the study is possible at any stage of the study without the need to state a reason and does not entail any negative consequences.

## Author Contributions

DS, CN, MD, SB, CL, WR, JG, and TS conception and design of the study. DS, ML, AM, and TS drafting of the manuscript. All authors critical revision, proofreading and approving of the submitted version.

## Funding

The study is funded by the Department of Anesthesiology, University Hospital Basel and by the Division of Clinical Psychology and Psychotherapy, Faculty of Psychology, University of Basel. After ethical approval our study was granted with an additional funding by the ProPatient research foundation of the University Hospital Basel. This funding source had no role in the design of this study. The funding source does and will not have any role during its execution, analyses, interpretation of the data, or decision to submit results either.

## Conflict of Interest

The authors declare that the research was conducted in the absence of any commercial or financial relationships that could be construed as a potential conflict of interest.

## Publisher's Note

All claims expressed in this article are solely those of the authors and do not necessarily represent those of their affiliated organizations, or those of the publisher, the editors and the reviewers. Any product that may be evaluated in this article, or claim that may be made by its manufacturer, is not guaranteed or endorsed by the publisher.
