# Supplementary material for: Open-Label Placebo Treatment for Acute Postoperative Pain (OLP-POP Study): Study Protocol of a Randomized Controlled Trial
Source: Front Med (Lausanne). 2021 Nov 5;8:687398. doi: 10.3389/fmed.2021.687398 (PMC8602681; doi:10.3389/fmed.2021.687398)
Supplement: Supplementary file 1 [file Data_Sheet_1.docx]

Supplementary Material

[1 Treatment Rationale OLP Group (English) 1](#_Toc83054251)

[2 Treatment Rationale OLP Group (German) 1](#_Toc83054252)

[3 Rationale Control Group (English) 2](#_Toc83054253)

[4 Rationale Control Group (German) 2](#_Toc83054254)

[5 Comprehensive Pain Assessment and Patients' Perception of Postoperative Pain Management 2](#_Toc83054255)

[6 Expectancy of Pain Relief 3](#_Toc83054256)

[7 Intervention Credibility Questionnaire 3](#_Toc83054257)

[8 Informed Consent (German) 4](#_Toc83054258)

# Treatment Rationale OLP Group (English)

The exact wording of the scripted treatment rationale provided alongside the first saline injection (T1), translated from German, is as following (cf. 2 for the German version):

“As part of the study you are participating in, you have been randomly assigned to the placebo group. This means that in addition to the standard treatment – the morphine pump and the other pain medication – you will receive an extra injection from us twice a day. These syringes do not contain painkillers, but saline solution. So, it's a placebo. We know from prior research that openly administered placebos are able to reduce pain clearly and in a clinically significant way. This has already been shown in 14 clinical studies since 2010. This reduction in pain through placebos is triggered, among other things, by the expectation of taking an effective medication. The expectation leads to the release of pain-regulating substances in the brain, which act against pain in the same way as morphine. This does not mean that you have to believe in it, but an open attitude is helpful. For these reasons, we assume that the injection with the placebo in addition to the morphine can help you to reduce the pain.”

At every subsequent placebo application (T2, T3, T4), the patient is reminded of the inertness of the injection and that we think that this injection might help with regulating pain. The exact wording of the reminder translated from German, is as following (cf. 2 for the German version):

“I will now give you another injection of the placebo. As you already know, this injection does not contain a painkiller, but a placebo, which we believe, in addition to morphine, can help you reduce the pain.”

# Treatment Rationale OLP Group (German)

Der exakte Wortlaut der Behandlungserklärung, die den Patienten direkt vor der ersten Injektion von Kochsalzlösung (T1) gegeben wird, ist wie folgt:

«Im Rahmen der Studie, an der Sie teilnehmen, sind Sie per Zufall der Placebogruppe zugeteilt worden. Dies bedeutet, dass Sie zusätzlich zur Standardbehandlung – also der Morphium-Pumpe und den anderen Schmerzmedikamenten – zwei Mal am Tag eine zusätzliche Spritze von uns verabreicht erhalten werden. Diese Spritzen enthalten kein Schmerzmedikament, sondern Kochsalzlösung. Es handelt sich also um ein Placebo. Wir wissen aus der Forschung, dass offen verabreichte Placebos in der Lage sind, Schmerzen deutlich und klinisch bedeutsam zu reduzieren. Dies konnte seit dem Jahre 2010 bereits in 14 klinischen Studien nachgewiesen werden. Diese Schmerzreduktion durch Placebos wird unter anderem durch die Erwartung, ein wirksames Medikament einzunehmen, ausgelöst. Die Erwartung führt im Gehirn zur Freisetzung von schmerzregulierenden Stoffen, welche auf dieselbe Art und Weise wie das Morphium gegen die Schmerzen wirken. D.h. aber nicht, dass Sie daran glauben müssen, jedoch ist eine offene Haltung hilfreich. Aus diesen Gründen nehmen wir an, dass die Spritze mit dem Placebo Ihnen zusätzlich zum Morphium helfen kann, die Schmerzen zu reduzieren.»

Bei jeder folgenden Placebo-Applikation (T2, T3, T4) werden die Patienten daran erinnert, dass die Injektion kein Schmerzmedikament enthaltet und dass wir denken, dass sie helfen kann, die Schmerzen zu reduzieren. Der exakte Wortlaut der Erinnerung der Behandlungserklärung ist wie folgt:

«Ich werde Ihnen nun erneut eine Spritze mit dem Placebo verabreichen. Wie Sie bereits wissen, enthält diese Spritze kein Schmerzmittel, sondern ein Placebo, von dem wir annehmen, dass es Ihnen zusätzlich zum Morphium helfen kann, die Schmerzen zu reduzieren.»

# Rationale Control Group (English)

After the randomization, participants allocated to the control group are informed about the treatment assignment as follows:

«As part of the study you are taking part in, you have been randomly assigned to the control group. This means that you will continue to receive the standard treatment consisting of the morphine pump and the other painkillers. The control group is important because this study is testing a new treatment and so we can test the effect of this new treatment compared to the standard treatment.»

# Rationale Control Group (German)

«Im Rahmen der Studie, an der Sie teilnehmen, sind Sie per Zufall der Kontrollgruppe zugeteilt worden. Dies bedeutet, dass Sie weiterhin die Standardbehandlung, bestehend aus der Morphium-Pumpe und den anderen Schmerzmitteln, erhalten werden. Die Kontrollgruppe ist wichtig, da diese Studie eine neue Behandlung testet und wir so die Wirkung dieser neuen Behandlung im Vergleich zur Standardbehandlung testen können.»

# Comprehensive Pain Assessment and Patients' Perception of Postoperative Pain Management

The IPO assesses patients pain experience and outcomes regarding the following patient reported aspects:

Pain severity and relief

Impact of pain on activity, sleep, and emotions

Side-effects of treatment

Ability to participate in pain treatment decisions

Use of non- pharmacological strategies

Chronic pain prior to surgery

The scale is administered at the following study visits:

- T-1; without items nr. 6, 9, and 10
- T0; without item number 13
- T3; without item number 13
- T5; without item number 13

# Expectancy of Pain Relief

Expectancy of pain relief are assessed separately for leg and back pain with a Likert-scale ranging from 0 - 10 (0 = no pain; 10 = worst pain imaginable respectively 0 = no need for pain relief at all; 10 = highest possible need for pain relief) at every study visit (i.e., T-1, T0, T1, T2, T3, T4, T5). The wording is as follows:

- Pre-Surgery (T-1): “In the following we would like to ask you about your expectations. On a scale from 0 (no pain) to 10 (most pain imaginable), how intense/severe will your lower back/leg pain be after full recovery from the successful surgery?”
- After Surgery (T0-T5): “In the following we would like to ask you about your expectations. On a scale from 0 (no pain) to 10 (most pain imaginable), how intense/severe will your lower back/leg pain be in the next 8 hours?”

# Intervention Credibility Questionnaire

The OLP-Group answers the following questions at T1 (given the answer possibilities “Not at all”, “Barely”, “A little”, “Fairly”, “Strong”, “Extreme”):

- «How credible did you find the explanation why the placebo treatment can work?”
- “How helpful did you find the explanation why the placebo treatment can work?”

At T5 the OLP-group is also asked: “During the study, did you believe that the syringes did not contain a pharmacologically active substance?”. Answer possibilities are:

- “Yes, I was sure they were "placebo" injections.”
- “I doubted that they were "placebo" injections.”
- “No, I didn't think they were "placebo" injections.”

If participants are not sure that the syringes contain placebo, they have to give open field answers: “Why did you have doubts?” respectively “Why didn't you believe they were "placebo" injections?”

# Informed Consent (German)

**Wirksamkeitsstudie eines offen verabreichten Placebos bei postoperativen Schmerzen (OLP-POP Studie)**

Open-Label Placebo Treatment for Acute Postoperative Pain: A Randomized Controlled Trial (OLP-POP Study)

Sehr geehrte Dame, sehr geehrter Herr

Wir möchten Sie anfragen, ob Sie an einer klinischen Wirksamkeitsstudie teilnehmen wollen. Im Folgenden wird Ihnen unser Studienvorhaben dargestellt:

1. **Ziel der Studie**

Wir untersuchen eine neue und innovative Behandlungsmethode für postoperative Schmerzen, die bei anderen Schmerzzuständen, wie beim Reizdarmsyndrom, chronischen Rückenschmerzen sowie bei akuten Schmerzen bei gesunden Personen, gute Wirksamkeit gezeigt hat. Bei dieser Behandlungsmethode handelt es sich um verabreichte Placebos (Scheinmedikament), d.h. die behandelten Personen erhalten ein Placebo und werden auch darüber informiert, dass sie ein Placebo erhalten. Das Ziel dieser Studie ist es, die klinische Wirksamkeit von offen verabreichten Placebos bei akuten Schmerzen in der post-operativen Schmerzbehandlung zu untersuchen.

1. **Auswahl**

PatientInnen, die innerhalb der Einschlusskriterien liegen und keines der Ausschlusskriterien erfüllen, können an der Studie teilnehmen. Die Kriterien sind wie folgt definiert:

**Einschlusskriterien:**

- PatientInnen bei denen eine Rückenoperation, genauer eine «Lumbale Wirbelkörper-versteifung» (LIF) geplant ist
- 18 Jahre oder älter
- Deutschsprachig oder sehr gute Deutschkenntnisse
- Fähigkeit, die Studie und ihre Ergebnisse zu verstehen
- Fähigkeit, eine informierte Einwilligung zur Studienteilnahme zu geben

**Ausschlusskriterien:**

- Bekannte chronische Schmerzen, die nicht mit den Beschwerden zusammenhängen, welche die geplante Operation zum Linderungsziel hat
- Bekannte neuromuskuläre Erkrankung
- Bekannte psychische Störungen
- Bekannter Drogen- oder massiver Alkoholkonsum
- Bekannte Nieren- oder Lebererkrankungen (GFR < 30)
- Bekannte Überempfindlichkeit oder Allergie gegen das Prüfpräparat (d.h. gegen Kochsalzlösung, NaCl 0.9%)
- Parallele Teilnahme an einer weiteren Studie mit Prüfpräparaten
- Mehr als 30 mg/Tag Morphinäquivalente präoperativ

1. **Allgemeine Informationen**

*Hintergrund der Studie:* Aus zahlreichen wissenschaftlichen Studien ist bekannt, dass Placebos eine nachweisbare und klinisch bedeutsame Wirkung haben. Dieser sogenannte «Placeboeffekt» ist speziell bei chronischen und akuten Schmerzzuständen zu beobachten und ist wissenschaftlich gut untersucht.

Es wird dabei davon ausgegangen, dass der Körper automatisch auf die Einnahme von Medikamenten reagiert und die dabei ausgelöste Schmerzlinderung durch die Freisetzung körpereigener Opioide vermittelt wird. Das heisst, der Körper produziert selber Stoffe, sogenannte Endorphine, die auf dieselbe Art und Weise wie sehr starke Schmerzmittel im Körper wirken.

Lange ging man davon aus, dass diese Placeboeffekte nur dann wirken, wenn die Studienteilnehmenden dachten, echte Medikamente zu erhalten und nicht wussten, dass sie Placebos einnehmen. Diverse Studien zeigen nun aber, dass Placebos auch wirken können, wenn die damit behandelte Person weiss, dass Sie ein Placebo verabreicht bekommen hat. So konnte zum Beispiel bei chronischen Schmerzzuständen, wie beim Reizdarmsyndrom oder chronischen Rückenschmerzen, bereits gezeigt werden, dass die offene Verabreichung eines Placebos effektiv zur Schmerzbehandlung beitragen kann. In der vorliegenden Studie wollen wir nun untersuchen, ob und wie offen verabreichte Placebos auch bei akuten postoperativen Schmerzen wirken.

*Organisation der Studie:* Die Studie wird durch die Abteilung der Schmerztherapie des Departments für Anästhesie und der spinalen Chirurgie des Universitätsspitals Basel in Zusammenarbeit mit der Abteilung für Klinische Psychologie und Psychotherapie der Psychologischen Fakultät an der Universität Basel durchgeführt. Wir planen 70 Patienten und Patientinnen in die Studie einzuschliessen.

Diese Studie wird so durchgeführt, wie es die Gesetze in der Schweiz vorschreiben. Ausserdem beachten wir alle international anerkannten Richtlinien. Die zuständige Kantonale Ethikkommission hat die Studie bereits geprüft und bewilligt. Eine Beschreibung dieser Studie finden Sie auch auf der Internetseite des Bundesamtes für Gesundheit: [www.kofam.ch](http://www.kofam.ch/) (Registrierungsnummer: SNCTP000003720). Zudem ist die Studie international auf der Seite [www.clinicaltrails.gov](http://www.clinicaltrials.gov/) registriert (Registrierungsnummer: NCT04339023).

1. **Ablauf**

- Studiengruppen: Es gibt zwei Studiengruppen, von denen Sie einer per Zufall zugeordnet werden: Eine Kontrollgruppe und eine Placebogruppe.
- Behandlung: Teilnehmende der Kontrollgruppe erhalten im Rahmen dieser Studie die Standardbehandlung für postoperative Schmerzen, welche sie auch ohne Studienteilnahme am Universitätsspital Basel erhalten würden. Teilnehmende der Placebogruppe erhalten zusätzlich zur Standardbehandlung zwei Mal pro Tag noch eine Spritze mit dem Placebo, wobei Ihnen bei der Verabreichung gesagt wird, dass Sie nun das Placebo erhalten.
- Befragungen: Unabhängig von der Studiengruppe werden wir Sie bitten, während vier ganzen Tagen Ihres Spitalaufenthaltes verschiedene Fragebogen auszufüllen (insgesamt 7). Dies beinhaltet zwei ausführlichere Befragung pro Tag (von einer Dauer von 10 Minuten) sowie in den ersten drei Tagen nach Ihrer Operation zusätzlich die zweistündliche Angabe der Schmerzintensität, die sie zum jeweiligen Zeitpunkt verspüren.
- Dauer der Studie: Die gesamte Studie dauert viereinhalb Tage und beginnt am Tag vor Ihrer Operation und endet am Morgen des dritten Tages nach Ihrer Operation.

Abbildung 1 (siehe nächste Seite) beschreibt den Ablauf der Studie sowie den damit verbundenen zeitlichen Aufwand für Patienten und Patientinnen, die am Tag vor der Operation eintreten. Alle Schritte, ausser die beiden orangen Kästchen (Operation & Beginn der Standardschmerzbehandlung), sind studienspezifisch. Für Patienten und Patientinnen, die erst am Tag der Operation selbst hospitalisiert werden, ändert sich dieser Ablauf dahingehend, dass die mündliche Studieninformation, die Überprüfung der Ein- und Ausschlusskriterien sowie das Einholen des schriftlichen Einverständnisses telefonisch am Tag vor der Operation durchgeführt werden. Ausserdem wird in diesem Fall die erste Befragung Zuhause am Computer ausgefüllt.

Es kann sein, dass wir Sie von der Studie vorzeitig ausschliessen müssen. Das kann zum Beispiel durch eine bewusste / unbewusste Verletzung des Studienprotokolls bedingt sein. Sollten sie aus medizinischen Gründen aus der Studie ausscheiden müssen, werden Sie ggf. zur Sicherheit nochmals fachärztlich untersucht.

*Abbildung 1.* Studiendesign und -ablauf bei Spitaleintritt am Tag vor der Operation.

1. **Nutzen**

Mit der Teilnahme an unserer Studie haben Sie die Möglichkeit, an einer neuartigen Behandlung teilzunehmen. Zusätzlich können die Resultate dieser Studie wichtig sein für das weitere Verständnis des Placeboeffektes bei akuten postoperativen Schmerzen sowie für die ethische Anwendung von Placebos im klinischen Alltag. Aufgrund des neuartigen Charakters der untersuchten Behandlung kann keine gesicherte Aussage über den zu erwartenden individuellen Nutzen für Teilnehmende beider Gruppen gemacht werden.

1. **Rechte**

Sie nehmen freiwillig teil. Wenn Sie nicht mitmachen oder später Ihre Teilnahme zurückziehen wollen, müssen Sie dies nicht begründen. Bei Abbruch der Studienteilnahme entstehen für Sie keinerlei negative Konsequenzen. Ihre medizinische Behandlung/Betreuung ist unabhängig von Ihrem Entscheid gewährleistet; eine allfällige Placebobehandlung endet mit Ihrem Entscheid, die Studie abzuschliessen. Sie dürfen jederzeit Fragen zur Studienteilnahme stellen. Wenden Sie sich dazu bitte an die Person, die am Ende dieser Information genannt ist.

1. **Pflichten**

Als TeilnehmerIn ist es notwendig, dass Sie sich an die notwendigen Vorgaben und Anforderungen der Studie durch den Prüfplan halten. Dies beinhaltet insbesondere das Ausfüllen der Befragungen zu den vorgegebenen Zeiten. Zudem bitten wir Sie, die Prüfperson über den Verlauf Ihrer Symptome zu informieren und neue Symptome, massive Symptomverschlechterung und sonstige relevante Änderungen im Befinden zu melden.

1. **Risiken und Belastungen für die Teilnehmenden**

Es sind keine studienspezifischen Risiken zu erwarten.

1. **Andere Behandlungsmöglichkeiten**

Sie müssen bei dieser Studie nicht teilnehmen. Wenn Sie nicht mitmachen, dann werden Sie die Standardbehandlung für akute postoperative Schmerzen erhalten.

1. **Ergebnisse aus dieser Studie**

Die Prüfperson wird Sie während der Dauer Ihrer Studienteilnahme über alle neuen Erkenntnisse informieren, die den Nutzen der Studie oder Ihre Sicherheit und somit Ihre Einwilligung zur Teilnahme an der Studie beeinflussen können. Sie werden die Information mündlich und schriftlich erhalten.

1. **Vertraulichkeit der Daten**

Für diese Studie werden Ihre persönlichen und medizinischen Daten erfasst. Nur sehr wenige Fachpersonen werden Ihre unverschlüsselten Daten sehen, und zwar ausschliesslich, um Aufgaben im Rahmen der Studie zu erfüllen. Bei der Datenerhebung zu Studienzwecken werden die Daten verschlüsselt. Verschlüsselung bedeutet, dass alle Bezugsdaten, die Sie identifizieren könnten (Name, Geburtsdatum), gelöscht und durch einen Schlüssel ersetzt werden. Die Schlüssel-Liste bleibt immer im Spital und wird an einem sicheren Ort aufbewahrt. Diejenigen Personen, die den Schlüssel nicht kennen, können daher keine Rückschlüsse auf Ihre Person ziehen. Bei einer Publikation sind die zusammengefassten Daten daher auch nicht auf Sie als Einzelperson rückverfolgbar. Ihr Name taucht niemals im Internet oder einer Publikation auf. Manchmal gibt es die Vorgabe bei einer Zeitschrift zur Publikation Einzel-Daten (sogenannte Roh-Daten) übermitteln zu müssen. Wenn Einzel-Daten übermittelt werden müssen, dann sind die Daten immer verschlüsselt und somit ebenfalls nicht zu Ihnen als Person rückverfolgbar. Alle Personen, die im Rahmen der Studie Einsicht in Ihre Daten haben, unterliegen der Schweigepflicht. Die Vorgaben des Datenschutzes werden eingehalten und Sie als teilnehmende Person haben jederzeit das Recht auf Einsicht in Ihre Daten.

Möglicherweise wird diese Studie durch die zuständige Ethikkommission oder durch die Universität Basel überprüft. Die Prüfperson muss eventuell Ihre persönlichen und medizinischen Daten für solche Kontrollen offenlegen.

1. **Rücktritt**

Sie können jederzeit aufhören und von der Studie zurücktreten, wenn Sie das wünschen. Die bis dahin erhobenen Daten werden danach noch verschlüsselt ausgewertet, weil das ganze Projekt sonst seinen Wert verliert.

Bei der Auswertung werden Ihre Daten vollständig anonymisiert, d.h. Ihre Schlüsselzuordnung wird vernichtet, so dass danach niemand mehr erfahren kann, dass die Daten und Proben ursprünglich von Ihnen stammten.

1. **Endschädigung für Teilnehmende**

Wenn Sie an dieser Studie teilnehmen, bekommen Sie dafür keine Entschädigung. Es entstehen Ihnen oder Ihrer Krankenkasse keine Kosten durch die Teilnahme.

1. **Haftung**

Das Universitätsspital Basel hat diese Studie veranlasst und ist für die Durchführung verantwortlich. Ausserdem haftet das Universitätsspital Basel für Schäden, welche Ihnen im Zusammenhang mit der getesteten Substanz oder den Forschungshandlungen (z.B. Untersuchungen) entstehen könnten. Die Voraussetzungen und das Vorgehen dazu sind gesetzlich geregelt.

Falls Sie einen Schaden erlitten haben, so wenden Sie sich bitte an die Prüfperson.

1. **Finanzierung der Studie**

Diese Studie wird vollständig durch das Department für Anästhesie (Schmerztherapie), das Departement für Spinale Chirurgie und der Abteilung für Klinische Psychologie und Psychotherapie der Fakultät für Psychologie der Universität Basel finanziert. Weitere externe Sponsoren sind nicht an der Finanzierung beteiligt.

1. **Kontaktpersonen**

Bei Fragen, Unsicherheiten oder Notfällen, die während der Studie oder danach auftreten, können Sie sich jederzeit an eine dieser Kontaktpersonen wenden.

*Dr. med Tobias Schneider*

Oberarzt am Department für Anästhesie / Schmerztherapie

Universitätsspital Basel, Spitalstrasse 22, CH-4031 Basel

Tel: +41613285165, E-mail: [tobias.schneider@usb.ch](file:///C:\Users\dsezer\Documents\tobias.schneider@usb.ch)

*Dilan Sezer, MSc*

Assistentin an der Abteilung für Klinische Psychologie und Psychotherapie

Fakultät für Psychologie, Universität Basel, Missionsstrasse 60/62, CH-4055 Basel

Tel: (+41) 061 207 61 89, E-mail: [dilan.sezer@unibas.ch](file:///C:\Users\dsezer\Documents\dilan.sezer@unibas.ch)

1. **Glossar von erklärungsbedürftigen Begriffen:**

**Was bedeutet „Placebo“?**
Manche Menschen, die ein Medikament bekommen, werden nicht durch das Medikament gesund, sondern erfahren alleine durch die die Zuwendung und Fürsorge des Arztes eine Besserung. Das kann man daran erkennen, dass es ihnen besser geht, selbst wenn sie ein sogenanntes Scheinmedikament bekommen. Dieses Scheinmedikament sieht aus wie ein echtes Medikament und ist auch gleich verpackt. Tatsächlich ist aber in diesem Scheinmedikament gar kein Wirkstoff. Man nennt es „Placebo“.
Manchmal behandelt man einen Teil der Teilnehmenden an einer klinischen Studie mit dem richtigen Medikament (mit dem Wirkstoff) und den anderen Teil mit einem solchen Placebo (ohne Wirkstoff). Dann kann man im Vergleich besser abschätzen, wie gut das Medikament tatsächlich wirkt oder ob die Besserung nur eintritt, weil die Menschen Zuwendung und Fürsorge erhalten. Manchmal entspricht die Besserung auch einfach dem natürlichen Verlauf der Krankheit.

**Was bedeutet „LIF“ ?**

LIF steht für “ lumbal interbody fusion”. Der englische Fachbegriff beschreibt die bei Ihnen vom Wirbelsäulenchirurg gewählte Operationstechnik zur Verbindung/Versteifung (Fusion) der Wirbelkörper. Wir haben uns entschieden nur PatientInnen in die Studie einzuschliessen die mit dieser Operationstechnik behandelt werden, um eine möglichst vergleichbare Gruppe an Patienten zu erhalten. Es ist uns wichtig zu betonen, dass die Wahl der operativen Behandlungsmethode in keinster Weise durch die Teilnahme an der Studie beeinflusst wird.
